# Breast stiffness, a risk factor for cancer and the role of radiology for diagnosis

**DOI:** 10.1186/s12967-023-04457-0

**Published:** 2023-08-30

**Authors:** Sofia M. Tarchi, Monica Pernia Marin, Md. Murad Hossain, Mary Salvatore

**Affiliations:** 1 Department of Radiology, Columbia University Irving Medical Center, New York, NY USA; 2 Department of Biomedical Engineering, Columbia University, New York, NY USA; 3 Department of Biomedical Sciences, Humanitas University, Via Rita Levi Montalcini 4, Pieve Emanuele, 20072 Milan, Italy

## Abstract

Over the last five decades, breast density has been associated with increased risk of developing breast cancer. Mammographically dense breasts are considered those belonging to the heterogeneously dense breasts, and extremely dense breasts subgroups according to the American College of Radiology’s Breast Imaging Reporting and Data System (BI-RADS). There is a statistically significant correlation between the increased mammographic density and the presence of more glandular tissue alone. However, the strength of this correlation is weak. Although the mechanisms driving breast density-related tumor initiation and progression are still unknown, there is evidence suggesting that certain molecular pathways participating in epithelial-stromal interactions may play a pivotal role in the deposition of fibrillar collagen, increased matrix stiffness, and cell migration that favor breast density and carcinogenesis. This article describes these molecular mechanisms as potential “landscapers” for breast density-related cancer. We also introduce the term “Breast Compactness” to reflect collagen density of breast tissue on chest CT scan and the use of breast stiffness measurements as imaging biomarkers for breast cancer screening and risk stratification.

## Introduction

The radiographic density of female breast tissue varies between individuals because of differences in the major tissue fractions’ relative abundance and radiological appearances [1–3]. These fractions, stroma, and epithelium, appear radio-dense (light) on mammograms while fat appears radiolucent (dark) [1, 4]. Radiologists subjectively and qualitatively estimate mammary breast density using the American College of Radiology’s Breast Imaging Reporting and Data System (BI-RADS) [3, 5, 6]. BI-RADS was implemented with the aim of standardizing and providing uniformity to radiological reports [7]. It offers a standardized terminology dictionary (the BI-RADS lexicon) through which to effectively communicate mammographic, ultrasound (US), and MRI image findings to clinicians and patients [7, 8]. More importantly, the BI-RADS system allows for the categorization of mammographic breast images into one of six breast density categories [5, 7, 9]. BI-RADS pioneered standardization in radiology reporting and was built to be fluid and evolve with scientific advancement [7]. As such, we believe it will continue to be used for the foreseeable future. Even so, the current version of BI-RADS presents limitations. For example, there is a lack of terminology describing non-mass lesions on US, masses on Digital breast tomosynthesis (DBT), findings on automated breast ultrasound (ABUS) coronal plane, and contrast-enhanced mammography examinations for which standardized terminology should be established [8]. Other shortcomings of the BI-RADS system are the fact that categories 3 and 4 are described differently on MRI compared to mammography and US, and the fact that interpretation of follow-up imaging examinations are not oriented towards the diagnosis of breast cancer that requires surgical treatment [8].

In 2016, Kerlikowske et al. used BI-RADS to estimate that 47% of US females between the ages of 40 and 74 are classifiable as having dense breast tissue [6]. Determinants of mammographic density variations have been found to be age, BMI, age at menarche, parity, age at first birth, breast-feeding, menopausal status, menopausal hormone therapy, family history of breast cancer, smoking, alcohol consumption, and physical activity [10, 11]. In 1976, Wolfe et al. were the first to recognize a strong positive correlation between the presence of densities and an increased risk of developing breast cancer [3, 12, 13]. Since then, considerable correlative data has been published in support of these findings and has determined this increase to be two to sixfold, a strength of association which is greater than that for most other established breast cancer risk factors, with the only exceptions being age and mutation of breast cancer genes [1, 3, 12, 14, 15].

Although statistically significant, the strength of the correlation between increased mammographic density and the presence of more glandular tissue alone is relatively weak [2]. For this reason, some point to stromal changes, such as significantly increased fibrillar collagen deposition, as being the major cause of breast radio-density [2, 14]. These observations are in keeping with the concept that stromal alterations might not always be ‘reactive’ but might sometimes play an initial ‘landscaping’ role in breast carcinogenesis [2]. Yet, the biological mechanisms driving breast density-related tumor initiation and progression remain uncertain [14].

### Molecular epithelial-stromal interactions, fibrillar collagen deposition, and invasive phenotype

An understudied aspect of the epithelial-stromal interaction is the fact that epithelial cells exist in a dynamic mechanical microenvironment, where dense collagenous stroma may play a significant role in governing cellular behavior [14, 16]. Traditionally, the extracellular matrix (ECM) has been viewed as an ultrastructure of molecules capable of providing structural and functional support [1, 2, 4]. However, it is now evident that in addition to providing integrity to stromal architecture, the matrix can also influence cellular apoptosis, gene expression, proliferation, differentiation, adhesion, and motility [1, 2, 4, 17]. In particular, the increased deposition of fibrillar collagen underlying mammographic densities has been shown to determine increased matrix stiffness and disrupt physiological mammary morphogenesis [16, 18]. Studies have pointed towards lysyl oxidase (LOX) mediated collagen crosslinking as the main contributor to stromal stiffening [16]. LOX are a family of enzymes having the capacity to remodel the ECM by catalyzing collagen and elastin crosslinks through the oxidation of their lysine and hydroxylysine residues [19–21]. LOX activity is required for the structural integrity of the ECM, increasing its tensile strength, and ultimately leading to stromal stiffening [16, 18, 19, 21–23]. Fibrogenic cells’ secretion of LOX proteins is induced through a Smad-dependent signaling cascade by transforming growth factor-β (TGF-β), a multifunctional cytokine that regulates ECM metabolism, microenvironmental homeostasis, and all stages of mammary gland development [21–24]. TGF-β influences mammary fibroblasts by increasing their expression of a variety of growth factors, cytokines, and more than a dozen ECM proteins involved in nearly every stage of collagen production and accumulation. Among these are procollagen-lysine 2-oxogluterate 5-deoxygenase (PLOD2) and prolyl-4-hydroxylase (P4HA3) which carry out posttranslational proline and lysine hydroxylations, chaperone proteins HSP47 and FKBP10 which prevent premature fibril formation, and meprin alpha/beta production which cleaves pro-collagen’s amino and carboxy terminal peptides, leading to the formation of fibrils [25]. Final transformations are carried out by additional TGF-β-induced proteins: fibronectin, LOX, plasminogen activator inhibitor 1 (PAI-1) and tissue inhibitor of metalloproteinases 1 and 3 (TIMP1, TIMP3) which inhibit collagen turnover [23–25]. Subsequently, biglycan and periostin control fibril packing and organization [25].

Even small increases in the microenvironment’s rigidity have been found to perturb tissue architecture by activating Rho GTPases and inducing collagen matrix contraction [14, 17, 26, 27]. Indeed, when activated, these molecular switches (in particular, small GTPase Rho-A) are charged with the regulation of Rho-associated protein kinase (ROCK) activity [26, 28]. In turn, ROCK phosphorylates myosin-II light chain (MLC2), the major motor protein responsible for generating cytoskeletal tension through its own contraction [26–28]. Conversely, it has been found that ROCK inhibition leads to myosin light chain 2 dephosphorylation and the consequent reduction in myosin contractility [26, 28]. Interestingly, researchers have observed significantly elevated levels of ROCK expression in tumor tissue compared to normal tissue. Moreover, the expression of ROCK is found to be notably higher in advanced stages of the disease and in patients with poor prognoses [29].

When cells become anchored to a non-compliant substrate such as this, the increase in resistance to cellular contractility or the application of external forces causes an increase in tension onto integrins—a family of transmembrane mechanotransducers involved in relaying ECM cues—ultimately leading to the activation of downstream signaling pathways [14, 17, 30]. Among these is the mechano-responsive recruitment of a variety of proteins—including talin, vinculin, paxillin, and focal adhesion kinase (FAK)—to the cytoplasmic domain of integrin receptors, with the aim of assembling focal adhesions (FA) [14, 30–33].

FAs are contact points between cells’ cytoskeletons and ECM proteins (collagen, fibronectin, or vitronectin) whose shape and dimensions are directly dependent on the magnitude of mechanical forces applied to the adhesion structures [14, 27, 28, 30, 31, 34]. These tight focal junctions work to anchor cells to their substrate, allowing for force transduction and the regulation of signaling cascades which ultimately determine the onset of invasive phenotypes [14, 31].

The central regulator of these cascades is the FAK enzyme, a widely expressed nonreceptor protein tyrosine kinase known to bind several signaling proteins implicated in signaling pathways regulating cell proliferation [35, 36]. Ample evidence derived from in vivo and ex vivo studies has shown that overexpression and activation of FAK enables tumor cells to survive in different environments and to colonize distal organs through their regulation of cell adhesion, migration, invasion, angiogenesis, and vascular permeability [37–39]. The first evidence that high FAK could be associated with an invasive phenotype was provided by Lewis et al. in 2001 [38]. Since then, molecular characterizations of tumor environments have allowed us to establish FAK’s role as part of a key transduction pathway implicated the progression of breast cancer in particular [39]. Indeed, FAK gene has been found to be amplified in over 40% of breast cancer specimens while being minimal in benign breast epithelium, thus linking its overexpression to tumorigenesis, progression, metastatic disease, an increased risk of recurrence, and reduced mammary cancer survival [37–40].

**Fig. 1:**
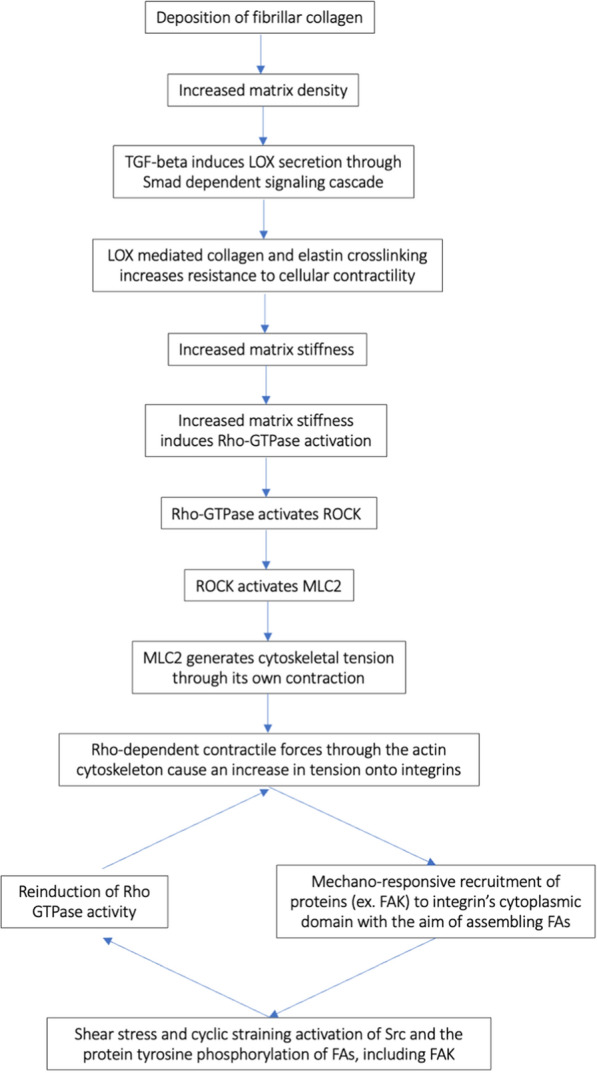
Flow chart of the molecular mechanisms involved in stroma stiffness. *TGF-beta* transforming growth factor-β, *LOX* lysyl oxidase, *ROCK* Rho-associated protein kinase, *MLC2* myosin-II light chain, *FAs* Focal adhesions, *FAK* focal adhesion kinase

Shear stress and cyclic straining of fibroblasts induce activation of Src family kinases (non-receptor tyrosine kinases known to influence cell proliferation, differentiation, and migration in a cell-autonomous manner) and the protein tyrosine phosphorylation of FAs, including FAK, p130 Cas, and paxillin [35, 41]. Among the outcomes of FAK’s phosphorylation is the reinduction of Rho GTPase activity. In so doing, a positive feedback loop that encourages additional FA creation, is formed [33]. This process is known as the Rho-FA-FAK loop and has been found to be chronically elevated in case of increased density and resistance to cell contractility [14, 33] (Fig. 1).

Alternatively, FAK’s stretch-induced signaling pathway may lead to mitogen-activated protein kinase (MAPK) activation and, thus, to the upregulation of proliferation and cell cycle related gene expression [14, 35]. Indeed, the phosphorylation of FAK’s major autophosphorylation site Tyr-397 residue creates a high-affinity binding site for the Src homology 2 (SH2) domain of Src family protein tyrosine kinases [14, 35, 36, 42]. The recruited Src mediates further phosphorylation of FAK on its Tyr-925 subunit, creating a binding site for the growth factor receptor bound protein 2 (Grb2)-son of sevenless (Sos) complex which leads to full activation of FAK as well as that of the Ras/MAPK pathway [35, 36, 42].

MAPK are a family of serine/threonine kinases made up of three subgroups: Extracellular-Signal Regulated Kinase (ERK), c-Jun NH_2_-terminal protein kinase (JNK), and p38 [35, 43]. These link extracellular signals to the nuclear machinery that controls fundamental cellular processes such as growth, proliferation, differentiation, migration, and apoptosis [14, 36, 43, 44]. This pathway makes use of Ras as its main molecular switch. This small GTPase protein, active when in the GTP-bound form, binds Raf kinase to promote its conformational change [44]. Activated Raf kinase subsequently phosphorylates and activates ERK kinase, which in turn phosphorylates and activates ERK [44].

**Fig. 2:**
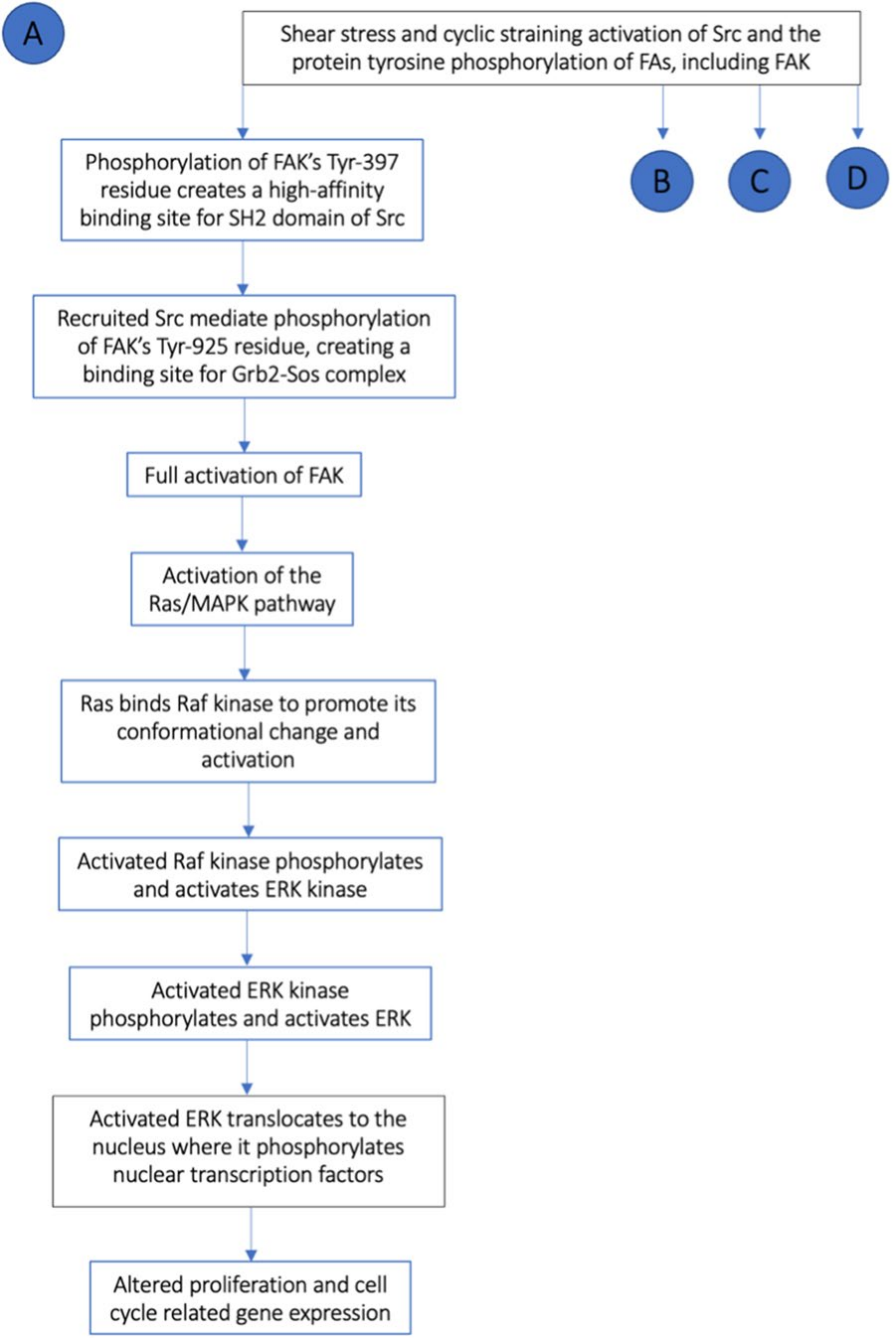
Flow chart representing the cascade of molecular events after FAK enzyme phosphorylation (A). *SH2* Src homology 2, *Grb2-Sos* growth factor receptor bound protein 2 (Grb2-son of sevenless (Sos), *ERK* Extracellular-Signal Regulated Kinase

FAK-regulated ERK phosphorylation and proliferation promote, depending on the particular cell type, proliferation, differentiation, survival, migration, angiogenesis, and chromatin remodeling [14, 43, 44]. ERK functions as a central regulator of the transcriptional response to increased matrix stiffness [14] (Fig. 2).

Indeed, western blot analysis has demonstrated a significant increase in ERK phosphorylation in cells cultured under high-density ECM conditions [14]. Furthermore, pro-growth signals in approximately one-third of all human cancers result from hyper-activation of the ERK pathway due to mutation/overexpression of its regulating molecules, such as Ras, receptor tyrosine kinases, or integrins [14, 44].

Activated ERK translocates from the cytoplasm to the nucleus where it is charged with regulating the mechanically-induced transcriptome shift through the phosphorylation of nuclear transcription factors [14, 43]. Subsequently, fibroblasts undergo upregulated proliferation, increased entry into and progression through their cell cycle, establishment and maintainance of a new invasive phenotype [14, 36, 43, 44].

### Effects on cell motility and migration

Among the effects FAK phosphorylation has on a highly dense ECM and its progression toward an invasive phenotype is the induction of increased cell motility [33]. This is achieved through the activation of three pathways: cytoskeletal rearrangement, FA dynamism, and nuclear signaling.

Cell migration is a coordinated membrane-based process that requires changes to the underlying cytoskeleton, rapid polymerization and stabilization of actin and microtubule filaments, as well as the formation and disassembly of cell adhesion sites [33, 45, 46]. In particular, it is FAK’s interactions with small guanosine triphosphate (GTP)-binding protein Rho and its effector, mammalian homolog of Diaphanous (mDia), that function to provide the molecular framework that supports directed cell motility [33, 46].

Through associations with Rho family GTPase-activating proteins (GAPs) and Rho guanine nucleotide-exchange factors (GEFs), FAK can phosphorylate α-actinin on Tyr12 resulting in the depolymerization of the actin cytoskeleton and the polymerization of actomyosin stress fibers [33, 45]. These newly formed bundles of polarized actin filaments present with fast growing plus ends and slow growing minus ends and have been shown to be involved in the generation of contractile force [33, 45]. At the cells’ leading edge, they are bundled into lamellipodia, filopodia, podosomes, invadopodia, and membrane ruffles (the dynamic process of lamellipodia folding back onto the cell body from which they previously extended) thus leading to variations in cell shape [33].

**Fig. 3:**
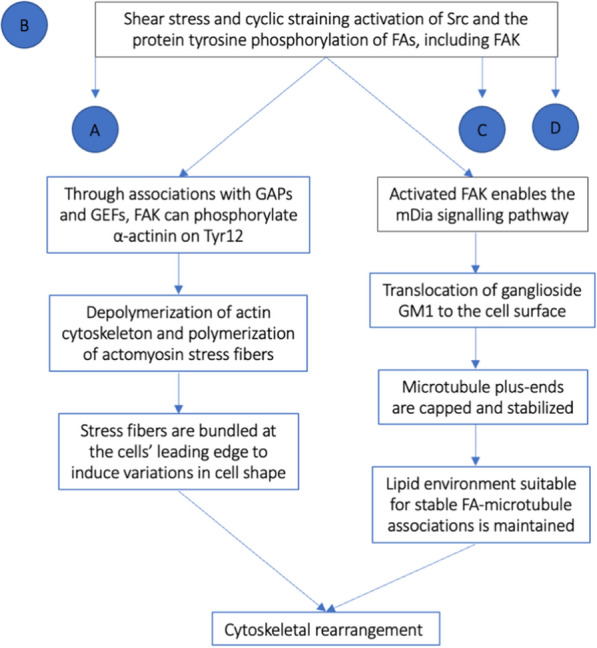
Flow chart representing the molecular mechanisms of cytoskeletal rearrangement secondary to FAK phosphorylation (B). *GTPs* GTPase-activating proteins, *GEFs* Rho guanine nucleotide-exchange factors

FAK also influences microtubule stabilization at the leading edge of migrating cells, important in the establishment and maintenance of cell polarity, through its Rho effector mDia signalling pathway which mediates the translocation of the lipid-raft marker, ganglioside GM1, to the cell surface [33, 46]. In doing so, microtubule plus-ends are capped, and a lipid environment which is suitable for localized stabilization of FA microtubule associations is maintained [33, 46]. In addition, cell motility is facilitated through the detachment of FAs from the trailing edge and their simultaneous formation in the cell’s leading edge [33, 45]. This coordinated cyclic disruption of FAs link to the actin cytoskeleton has been shown to be essential for efficient cell migration [45, 47] (Fig. 3).

Our understanding of the precise mechanism controlling FAs’ assembly and disassembly is currently limited, however, recent studies have shown FAK and microtubule assembly play central roles in this process [45, 47–49]. It has been speculated that the growth of microtubules (as detailed above) can promote focal adhesion dissolution by serving as tracks across which to deliver key disassembly factors [48, 49].

Growing microtubules accumulate at their plus ends multiple structurally unrelated molecules collectively termed microtubule plus end tracking proteins [49]. The most conserved and ubiquitous microtubule plus end tracking proteins are end binding proteins (EB) [49]. Mammalian cells express three EB proteins—EB1, EB2, and EB3—that share substantial sequence similarity and can all track the plus ends of growing microtubules [48, 49].

**Fig. 4:**
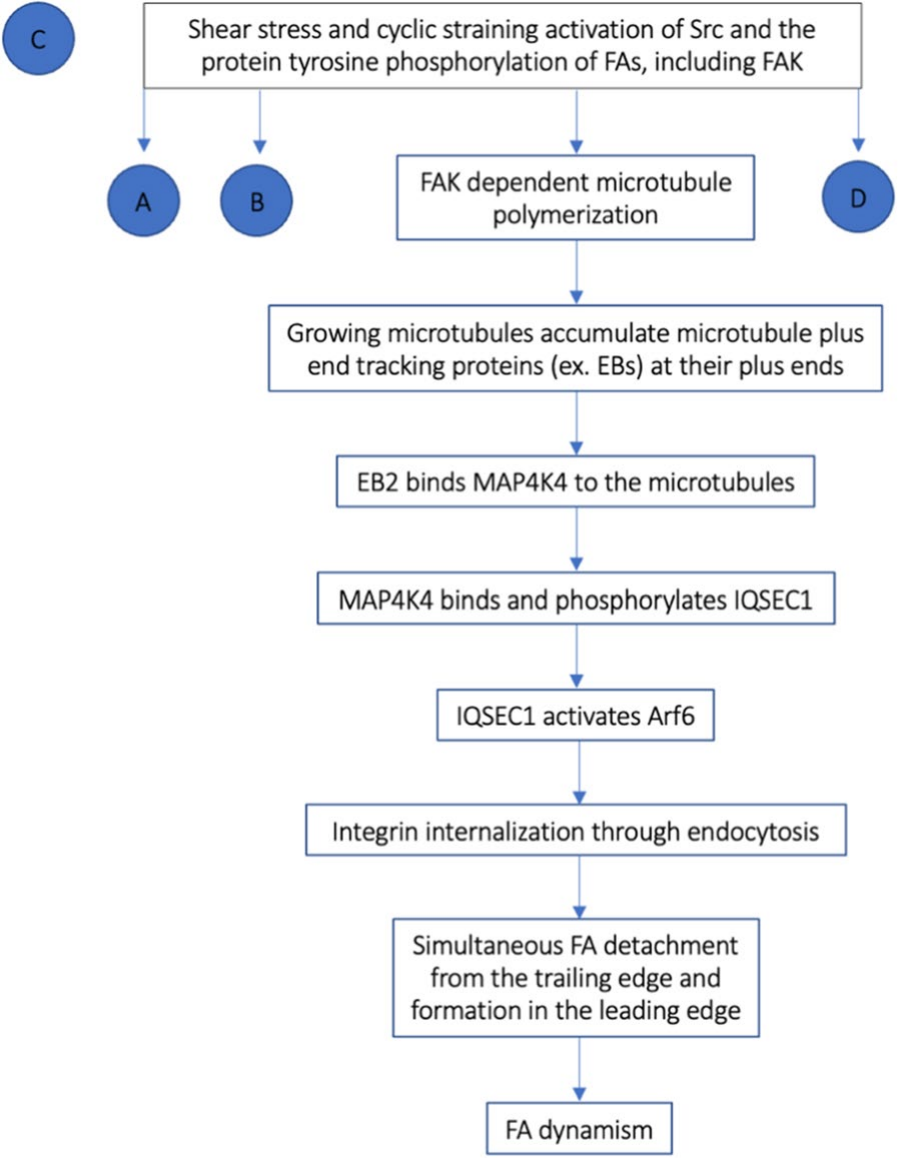
Flow chart of the effect of FAK phosphorylation on microtubule polymerization (C). *EB2* end binding protein 2, *MAP4K4*: mitogen-activated protein 4 kinase 4, *IQSEC1* IQ motif and SEC7 domain-containing protein 1, *Arf6* ADP-ribosylation factor 6, *FA* focal adhesion

Among the three proteins, EB1 and EB3 are usually considered to be the master regulators of microtubule dynamics by promoting microtubule growth and suppressing catastrophe [48, 49]. Instead, EB2 plays an essential role in the regulation of focal adhesion dynamism and, in turn, cell migration due to its interaction with a mitogen-activated protein 4 kinase 4 (MAP4K4) [48, 49]. MAP4K4 is a microtubule-dependent factor and FA regulator that associates with microtubules via its interaction with EB2 [48, 49]. It can be delivered to the focal adhesion sites and promote their disassembly by binding and phosphorylating IQ motif and SEC7 domain-containing protein 1 (IQSEC1), a guanine nucleotide exchange factor specific for ADP-ribosylation factor 6 (Arf6), a member of the ADP ribosylation factor family of GTP-binding proteins. In turn, IQSEC1 interacts and activates Arf6, thus promoting integrin internalization through endocytosis [48, 49] (Fig. 4).

**Fig. 5:**
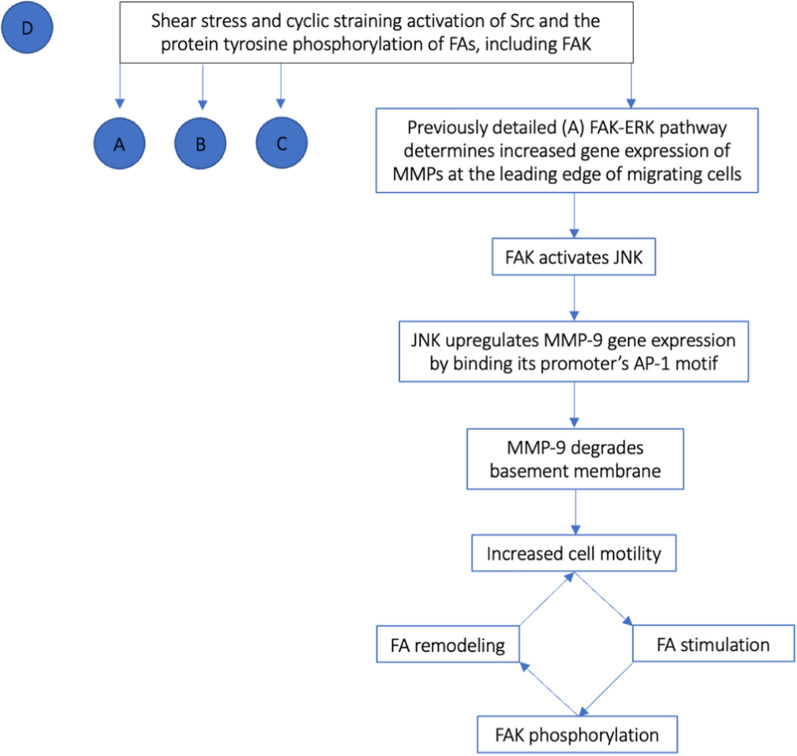
Flow chart of the effect of FAK phosphorylation in the upregulation of gene expression of matrix metalloproteinases (MMPs) leading to degradation of basement membranes increasing cell mobility and perpetuating FA remodeling (D). *JNK* c-Jun NH_2_-terminal protein kinase

**Fig. 6:**
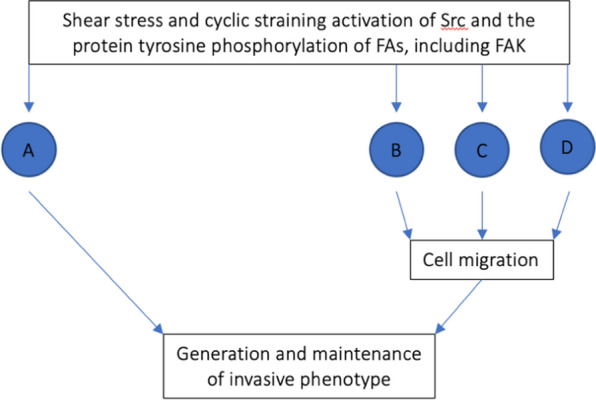
FAK phosphorylation activates multiple molecular cascades (A, B, C, and D) that favor cell migration and breast carcinogenesis

Finally, FAK phosphorylation also induces increased cell motility in highly dense ECM through nuclear signaling. Indeed, the previously detailed FAK-ERK pathway through which gene expression is altered has also been found to upregulate the transcription and activation of proteolytic enzymes, such as matrix metalloproteinases (MMPs), at the leading edge of migrating cells [50–53]. The increased expression of MMP-9, in particular, has been found to be associated with a metastatic tumour cell phenotype [33, 52, 53]. Especially, through the activation of JNK, active FAK can promote MMP-9 gene expression at its promoter’s AP-1 motif [51, 53]. In so doing, the targeted degradation of basement membrane is promoted and cell spreading, and growth are facilitated [51–53]. In turn, increased cell motility is sensed by FAs, provoking FAK phosphorylation, and consequent focal adhesion remodeling (as detailed above) thereby further increasing cell motility [33, 51] (Figs. 5, 6).

### Future directions

**Fig. 7:**
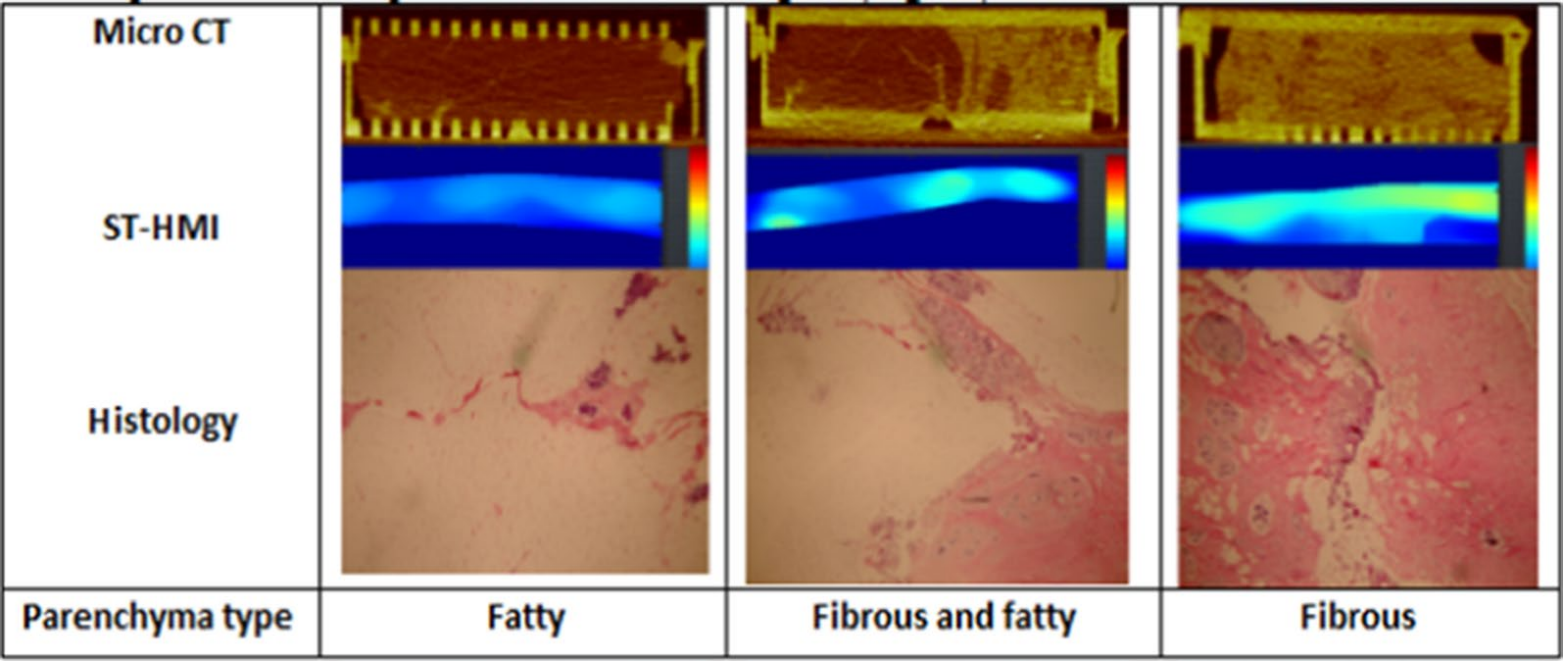
Three non-cancerous breast specimens which on visual inspection could be classified broadly as fatty, mixed, and fibrous. Micro CT correlates well with the extent of fat and fibrous tissue found on histology. Areas that contain fat, low Hounsfield Units (HU) on CT have slow ST-HMI-derived group velocity (ms^−1^) and tissue that contains fibrous tissue with high HU on CT have fast ST-HMI-derived group velocity (ms^−1^), and mixed fat and fibroglandular tissue (medium HU) has a variable velocity that corresponds to extent of collagen and its stiffness. Fatty parenchyma type presents with highly fatty (white) histology with minimal fibrous histology (red), slow ST-HMI-derived group velocity (blue), and low HU on CT (dark). Fibrous and fatty parenchyma type presents with mixed amounts of fatty (white) and fibrous (red) histology, average ST-HMI-derived group velocity (light blue/green), and average HU on CT (mixed amounts of dark and light). Fibrous parenchyma type presents with highly fibrous (red) histology with minimal fatty histology (red), fast ST-HMI-derived group velocity (yellow), and high HU on CT (light)

Based on the preceding, an imaging biomarker for breast stromal stiffness would transform breast cancer screening because it would not only inform who was at greatest risk but the area of the breast that is most vulnerable. Single transducer–harmonic motion imaging (ST-HMI) is an ultrasound elastography method that estimates the mechanical properties (e.g., elasticity and viscosity) of tissues [54]. ST-HMI uses a clinical ultrasound system with an imaging transducer to generate an amplitude modulated—acoustic radiation force (AM-ARF, i.e. force due to propagating long ultrasound pulse) for oscillating tissue at a particular frequency. To interrogate mechanical properties, ST-HMI assesses both displacements ‘on-axis’ to AM-ARF [55] and phase and group velocities ‘off-axis’ to AM-ARF [56]. Displacements provide qualitative mechanical properties of the tissue whereas phase and group velocities provide quantitative mechanical properties. For example, group velocity (V) is related to Young’s modulus (E) as E = 3ρV^2^ where ρ is the density of the tissue. Figure 7 demonstrates three non-cancerous breast specimens which on visual inspection could be classified broadly as fatty, mixed, and fibrous. We obtained micro-CT imaging using microCT system (U-CT, MILabs, Netherlands) and the following parameters: Filter—250 μM Aluminum foil, Tube voltage—30 kV, current—0.16 mA, exposure—240 ms, 0.5°, 1 projection/step, Voxel size 0.1 × 0.1 × 0.1 mm. Fibroglandular tissue was gold and fat was rust-colored on CT allowing more accurate quantification of the character of the tissue. Micro CT correlates well with the extent of fat and fibrous tissue found on histology. Furthermore, areas that contain fat, low Hounsfield Units (HU) on CT have slow ST-HMI-derived group velocity and tissue which contains fibrous tissue with high HU on CT has fast ST-HMI-derived group velocity, and mixed fat and fibroglandular tissue (medium HU) has a variable velocity that corresponds to extent of collagen and its stiffness. Within the fibrous and fatty tissue section, the fibrous section (right side) has a higher group velocity and higher HU compared to the fatty section (left side).

**Fig. 8:**
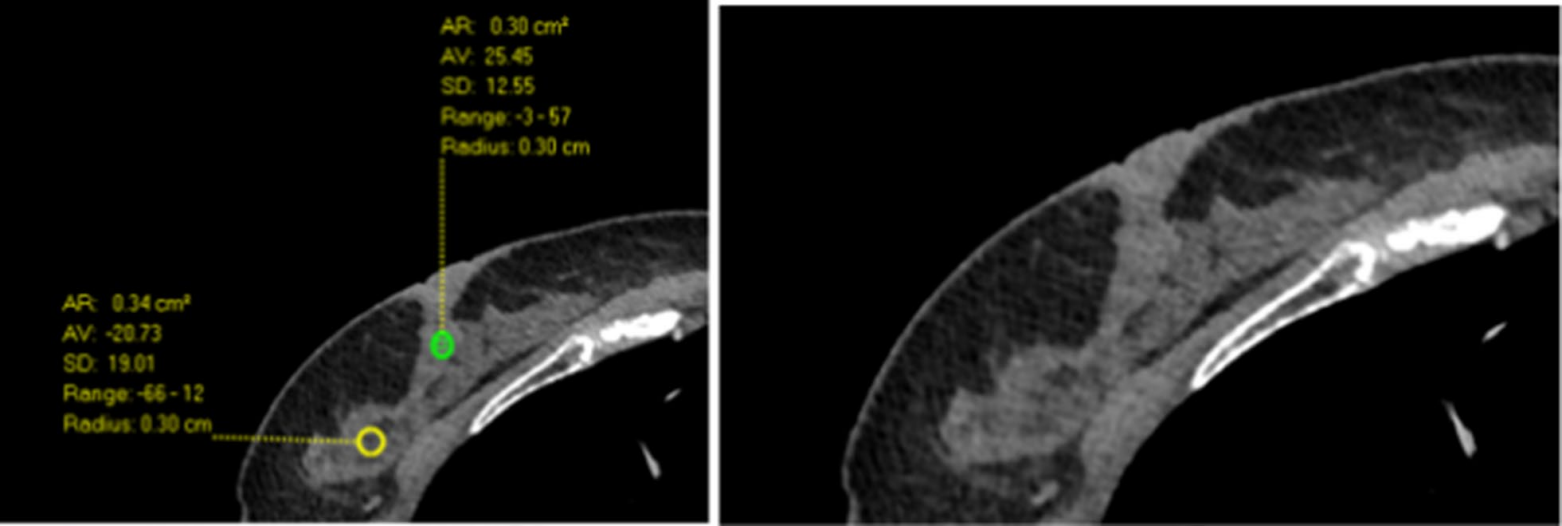
Axial chest CT image measuring 2 regions of interest in the right breast. The yellow circle has a mx HU of 12 and therefore is less dense that the green circle with HU of 57 which is denser

We would like to coin the term “*Breast Compactness*” a reflection of collagen density which can be quantified on chest CT by measuring the maximum HU of the breast parenchyma in a 3mm region of interest. The Hounsfield unit measures radio-density; denser tissue has more positive numbers and appear lighter; less dense tissue has more negative numbers and appears darker [57]. The HU max is a surrogate biomarker for the extent of collagen and likely its organization as demonstrated by ST-HMI and CT, it varies from one area of the breast to another as seen in (Fig. 8). Further research is necessary to evaluate the usefulness of “*Breast Compactness*” in not only predicting those at increased risk for breast cancer but also for following-up the benefits of interventions to decrease risk.

## Conclusion

The cause of breast radio-density and its relationship with breast carcinogenesis is not fully understood. Evidence suggests that fibrillar collagen deposition may be responsible for the increased extracellular matrix density and stiffness that could explain the presence of breast radio-density as well as a favorable microenvironment for the origination, proliferation, and invasion of cancer cells. At the molecular level, several cascades have been identified as important regulators of the epithelial-stromal interaction which is determinant for the configuration of the ECM. The FAK enzyme is a widely expressed nonreceptor protein tyrosine kinase and the main regulator of these cascades. It ultimately causes alterations in the proliferation and cell cycle gene expression through the ERK-mediated phosphorylation of nuclear transcription factors. FAK phosphorylation is also the starting point of molecular pathways that cause cytoskeletal rearrangement through the stabilization of the FA-microtubule associations and the polymerization of actomyosin stress fibers at the cellular leading edge inducing cellular shape variations. In addition, FAK dependent microtubule polymerization also loosens focal adhesions. The combination of focal adhesion dynamism and the basement membranes degradation by FAK-dependent MMP-9 gene upregulation leads to increased cell motility and migration generating and maintaining the invasive phenotype of breast cancer.

We obtained ST-HMI, an ultrasound elastography method that estimates the elasticity and viscosity of tissues and compared it to micro-CT imaging [54]. ST-HMI uses a clinical ultrasound system with an imaging transducer to generate an AM-ARF for oscillating tissue at a particular frequency. It assesses displacements ‘on-axis’ to AM-ARF to provide qualitative mechanical properties of the tissue [55] as well as phase and group velocities ‘off-axis’ to AM-ARF with the aim of providing quantitative mechanical properties [56]. Instead, micro-CT imaging evaluates HU, a measure of radiodensity—denser tissues appear lighter and have more positive numbers, less dense tissue appears darker and has more negative numbers [57]—and allows for accurate quantification of the character of the tissue, correlating well with the extent of fat and fibrous tissue found on histology.

In particular, we found that areas that contain fat present low HU on CT and have slow ST-HMI-derived group velocity while areas that contain fibrous tissue present high HU on CT and have fast ST-HMI-derived group velocity. Thus, the HU max could serve as a surrogate biomarker for the extent of collagen and likely its organization. To this end, we have coined the term “*Breast Compactness*”, a reflection of collagen density which can be quantified on chest CT by measuring the maximum HU of the breast parenchyma in a 3mm region of interest. *Breast compactness* could become an imaging biomarker for breast stromal stiffness with the potential to improve current breast cancer screening practices and cancer risk stratification as it would not only inform who was at greatest risk but the area of the breast that is most vulnerable.

Further research is necessary to evaluate the usefulness of this prospective biomarker in not only predicting those at increased risk for breast cancer but also for following-up the benefits of interventions to decrease risk. More specifically, studies investigating breast compactness or the FAK pathway as a target for therapeutic intervention should be carried out. The inhibition of FAK overexpression as a potential cancer treatment strategy has been a focus of oncological research, and several FAK inhibitors have been proposed [58, 59]. Current strategies entail competitive or allosteric inhibition of FAK kinase activity as well as direct interference with FAK autophosphorylation which spares off-target effects [58].

## Data Availability

Data sharing not applicable to this article as no datasets were generated or analyzed during the current study.
